# The Development and Co‐Production of a Caregiver Coping Resource for Pediatric Inflammatory Bowel Disease and Autoimmune Liver Disease

**DOI:** 10.1002/lrh2.70061

**Published:** 2026-02-17

**Authors:** Jennie G. David‐Rodgers, Christina E. Holbein, Hannah McKillop, Maria E. Lester, Victoria Levine, Ildiko Mehes, Heidi C. Riechel, Jane R. Weyer, ImproveCareNow Learning Health System, Autoimmune Liver Disease Network for Kids Learning Health System

**Affiliations:** ^1^ Pediatric Psychology and Neuropsychology Nationwide Children's Hospital Columbus Ohio USA; ^2^ Psychiatry and Behavioral Sciences Children's Hospital of Philadelphia Philadelphia Pennsylvania USA; ^3^ Nemours Children's Health Orlando Florida USA; ^4^ Children's Hospital of Philadelphia Philadelphia Pennsylvania USA; ^5^ Children's Healthcare of Atlanta GI Care for Kids Atlanta Georgia USA; ^6^ Cincinnati Children's Hospital Medical Center Cincinnati Ohio USA

**Keywords:** caregivers, pediatrics, psychosocial, quality improvement

## Abstract

**Objective:**

Caregivers of children with chronic conditions have mental health needs, with no known resources for caregiver coping in pediatric Inflammatory Bowel Disease (IBD) and autoimmune liver disease (AILD), which can be co‐morbid. Quality improvement (QI) has previously co‐produced resources within Learning Health Networks (LHNs). This QI work sought to co‐produce a caregiver coping resource for caregivers of children with IBD and/or AILD.

**Methods:**

A multidisciplinary QI team of caregivers and psychosocial clinicians applied QI methodology to iteratively develop a caregiver coping resource. This work took place within two connected LHNs, ImproveCareNow (pediatric IBD) and Autoimmune Liver Disease Network for Kids (AILD).

**Results:**

Over 1.5 years, a multidisciplinary QI team of eight caregivers and four psychosocial clinicians co‐produced a caregiver coping resource. The formatted caregiver coping resource is 164 pages long with sections including caregiver coping (e.g., anxiety), caregiver support of child coping (e.g., discussing emotions), special considerations (e.g., surgery in IBD), and logistical topics (e.g., navigating insurance). The resource integrates caregiver and psychosocial clinician quotes and reputable resources. The resource is freely available and information about how to access the resource is included in the manuscript text.

**Conclusion:**

While caregivers of children with IBD and/or AILD have known coping needs, caregiver‐focused coping resources are urgently needed. A multidisciplinary team of caregivers and psychosocial clinicians within LHNs co‐produced such a resource that is freely accessible. This QI work demonstrates the collaborative potential to build caregiver‐focused resources for all families to benefit. Future work is needed to understand the clinical use of this resource as well as the impact of this resource for caregivers of children with IBD and/or AILD.

## 1 Introduction

### 1.1 Problem Description

The extant literature demonstrates that caregivers of children with chronic conditions, including Inflammatory Bowel Disease (IBD) and autoimmune liver disease (AILD), can experience psychosocial concerns like anxiety or depressed mood [1, 2]. However, resources related to coping with pediatric IBD and AILD are geared toward children and adolescents, with limited resources focused on caregiver coping. Within the ImproveCareNow (ICN) Learning Health Network (LHN), there were resources designed for pediatric IBD patients though none were designed specifically for caregivers [3]. Additionally, a cohort of patients has co‐morbid IBD and AILD diagnoses [4], which present cross‐cutting and unique coping needs for caregivers of children and adolescents with IBD and AILD (e.g., coping with unpredictable health needs, the potential role of liver transplant in AILD). Consequently, there was a pressing clinical need to develop a resource focused on caregiver coping in pediatric IBD and AILD.

### 1.2 Available Knowledge

In August 2024, the US Surgeon General issued an advisory about rising concerns for parental mental health and the need for additional supports and services for American caregivers [5], including a call for healthcare systems to provide additional support for caregivers of children with health needs. Salley et al. [6] recently advocated for formalized standards of care targeting the mental health needs of caregivers of youth with chronic medical conditions, including screening programs and behavioral health interventions. Indeed, caregivers of children with chronic medical conditions, including IBD and AILD, may be particularly at risk for experiencing stress and unmet psychosocial needs [7, 8, 9]. Specific to IBD, caregivers experience stress around navigating the impact of IBD on family and social life, managing difficult conversations with the medical team, coping with intense emotions about their child's condition, and socioeconomic burden [10, 11]. Caregivers may experience more significant caregiving‐related stress around the time of their child's diagnosis; it has been proposed that caregiver‐specific resources focused on parenting stress and coping may be particularly beneficial within the first year [12]. Providing avenues for additional caregiver support is critical due to the established associations between caregiver stress and psychosocial functioning and child outcomes, including depressive symptoms, disease activity, and health‐related quality of life [12]. While less is known about the psychosocial needs of parents of youth with AILD, recent guidance calls for increasing assessment and supports specifically for caregiver psychosocial needs [13].

Social support from other caregivers of children with a chronic medical condition has been shown to have positive effects on caregiver coping and psychological well‐being [14]. Caregivers of youth with chronic medical conditions have advocated for more accessible, high‐quality social support resources, such as parent‐to‐parent connections, support groups, and family‐based groups [15, 16, 17]. However, families may face challenges in accessing caregiver support resources due to a lack of available resources or programs affiliated with their medical center and logistical difficulties attending scheduled meetings or events. While many caregivers may benefit from seeking information and support from condition‐specific online forums and social media [18], content may not be evidence‐based and may reflect skewed views and experiences [19, 20]. Our team sought to create an educational resource targeting caregiver well‐being and coping that would be freely available to caregivers at any time.

ICN has an established history of developing educational and supportive materials for pediatric IBD that leverage both patient perspectives and psychosocial expertise, and they have created a formalized process for generating such resources [3]. Previously, psychosocial clinicians and pediatric patients with IBD have created toolkits to support adjustment related to various aspects of living with IBD, including coping with an ostomy [21], transferring to adult GI care [22], disordered eating, and school accommodations.

### 1.3 Rationale

Facilitating meaningful engagement from primary stakeholders (patients and caregivers) is an important tenet of LHNs like ICN and A‐LiNK. Previous research related to patient and caregiver engagement has reflected diverse engagement opportunities, ranging from relatively limited and shallow engagements where patients or caregivers may be asked to give feedback on a project that is already developed to more in‐depth and arguably meaningful engagement, such as co‐producing resources for the patient community [23]. Engagement has been previously conceptualized as a “ladder of engagement” [24] and more recently proposed “levels” of engagement [23]. The QI initiative that is summarized in this manuscript incorporates models of the ladder of engagement and levels of engagement, as well as conceptualizing meaningful engagement that promotes “bottom up” generation of resources for the illness community by the illness community [21]. These engagement models have also been reflected in co‐production throughout ICN in developing, creating, and disseminating other resources for the illness community [3, 21, 23].

The partnership between psychosocial clinicians and caregivers was a key component in the resource development process, which is described in greater detail below. It is well‐established that patient and family engagement in the design, delivery, and assessment of health services can have direct benefits for enhancing clinical services and resources [23, 25]. While both psychosocial clinicians and caregivers created and revised content for the caregiver coping resource described in this manuscript, each role had specific objectives: caregivers provided guidance from their lived experiences as parents of pediatric patients with IBD and AILD and provided a lens of community and social support to caregiver readers; psychosocial clinicians contributed content based on clinical training, clinical experiences, and knowledge of evidence‐based care.

### 1.4 Specific Aims

The specific aims for this QI initiative centered on (1) the co‐production of a caregiver coping resource for caregivers of pediatric patients with IBD and/or AILD by a multidisciplinary team of caregivers and psychosocial clinicians, and (2) writing and disseminating resource development process through a peer‐reviewed journal to demonstrate how this meaningful collaboration is possible, can result in tangible resources for illness communities, and may serve as a roadmap for other pediatric illness communities to develop their own caregiver‐focused coping resources.

## 2 Methods

### 2.1 Context

ICN is a LHN dedicated to pediatric IBD, with 101 hospitals primarily in the United States actively participating in the LHN [26, 27]. As a LHN, ICN supports and fosters collaboration amongst diverse stakeholders including physicians, psychosocial clinicians (psychologists and social workers), patients, and caregivers; co‐production of resources has been previously described in the extant literature [3, 21, 22]. ICN has various stakeholder groups, including the Parent Family Advisory Council (PFAC) and the Social Work and Psychology (SWAP) group, which supports community building and knowledge sharing through routine meetings. All stakeholder groups are invited and welcome to attend the twice‐yearly network‐wide conferences as another opportunity to support and showcase collaboration across ICN. The culture of ICN has been previously described [23] and promotes innovation and novel collaboration intra‐ and inter‐stakeholder groups. The Autoimmune Liver Disease Network for Kids (A‐LiNK) is a LHN supported by ICN and is comprised of 12 hospitals (all of which are also ICN sites) [28]. Given the co‐morbidity between autoimmune liver disease (AILD) impacting a cohort of patients with IBD [4, 29], these LHNs work collaboratively together for patients with co‐morbid IBD and AILD diagnoses. In summary, ICN and A‐LiNK collectively care for pediatric patients with IBD, AILD, and co‐morbid IBD and AILD diagnoses.

The SWAP group is comprised of psychosocial clinicians (psychologists and social workers) from ICN, with quarterly virtual meetings and frequent collaboration with other entities within ICN (e.g., co‐producing a podcast series on mental health with ICN's Patient Advisory Council members). Following a clinical care experience where one psychologist in the SWAP was interacting with a family and reflecting on the limited caregiver‐focused resources, the psychologist (JGD) reached out to the PFAC leadership (HR, IM) to share an idea to create a caregiver‐focused coping resource. Unbeknownst to SWAP, the PFAC was independently having internal discussions about projects supporting caregiver mental health and creating such a resource. Additionally, A‐LiNK was also independently reflecting on their goals and strong interest in caregiver mental health. As described below, the shared context of LHNs (ICN and A‐LiNK) facilitated collaboration and engagement in a QI initiative across stakeholder groups.

### 2.2 Intervention

Within the context of ICN and A‐LiNK, the SWAP and PFAC were well‐known to each other with previous collaborations as well as meeting in‐person at ICN conferences where all community stakeholders are present (e.g., physicians, psychosocial clinicians, patient advocates, caregiver advocates). As described above, a clinical experience led to emerging and organic discussions between SWAP and PFAC, and the growing team proceeded to share this developing opportunity within the respective groups as well as posting the opportunity to the larger ICN network per network‐level communications. Through this process, 11 caregivers and six psychosocial clinicians (four psychologists, two social workers) initially self‐identified as interested; as the process of creating the caregiver resource continued, the team reduced to eight caregivers (including M.E.L., V.L., I.M., H.R., J.R.W.) and four psychosocial clinicians (three psychologists, one social worker; including J.G.D., C.E.H., H.M.). The primary reasons for the three caregivers and two psychosocial clinicians withdrawing from the QI initiative related to limited time and life or career transitions (e.g., parental leave). Please see Table 1 for data related to the composition of our team.

**TABLE 1:** Quality improvement team demographic information.

Caregivers	
Caregiver role	
Mom	100% (*n* = 8)
Caregiver age	
*M* age in years	51.7 (range: 28–63)
Caregiver ethnicity	
Hispanic	12.5% (*n* = 1)
Non‐Hispanic	87.5% (*n* = 7)
Caregiver race	
White	75% (*n* = 6)
Multiracial	12.5% (*n* = 1)
Missing	12.5% (*n* = 1)
Children's diagnoses	
IBD Only	62.5% (*n* = 5)
*M* age of child at IBD diagnosis in years	11.8 (range: 2–23)
IBD and AILD	37.5% (*n* = 3)
*M* age of child at AILD diagnosis in years	19.3 (range: 10–25)
Psychosocial clinicians	
Psychosocial clinician role	
Psychologist	75% (*n* = 3)
Social worker	25% (*n* = 1)
Psychosocial clinician age	
*M* age in years	33.25 (range: 26–38)
Psychosocial clinician race	
White	75% (*n* = 3)
Asian	25% (*n* = 1)
Education level	
Doctoral degree	75% (*n* = 3)
Master's degree	25% (*n* = 1)
Years practicing as a licensed psychosocial clinician	
*M* years	3.94 (range: 2–5.75)

Our QI team began virtual conference calls in March 2023 with the goal of collaboratively clarifying our goals for creating a caregiver‐focused resource. Our QI team created a Key Driver Diagram to guide the QI work and implemented the iterative approach utilized in the development of other ICN toolkits [3, 21]. Our team met every 1–2 months through the end of 2023 as we refined our vision of the caregiver coping resource, finalized the topics of interest to include (e.g., coping with a new diagnosis), and caregivers and psychosocial clinicians self‐identified which topical sections they wanted to write from the list of finalized topics; allowing caregivers and psychosocial clinicians to self‐identify which topical section(s) they wanted to contribute to encouraged team members to have agency over which topic(s) they felt passionate and comfortable writing about as well as supporting team members in choosing writing tasks that fit within their volunteer bandwidth (i.e., choosing multiple topics if the individual had more time to dedicate to writing). All team members were directed to self‐identify interest in as many topics as desired with no limit to the number of team members who could contribute on a given topic (i.e., no maximum contributor limit for each topic). Throughout 2023, our team met seven times with agendas and minutes recorded for each meeting. Writing the topical sections for the caregiver coping resource was completed asynchronously and via a cloud‐based application, with each team member completing their self‐selected writing assignments when this volunteer work was feasible within their schedules. Throughout the writing process, the caregivers wrote their topical sections first and then the psychosocial clinicians wrote their sections to ensure that the caregiver voice was centered in the process.

When our team had a full first draft of the caregiver coping resource in June 2024, the resource was then iteratively reviewed by QI team members and ICN Communications staff for organization, redundancy, and missing context. At this time, our team recognized the lack of non‐mother caregiver voices and the caregiver members directly asked non‐mother caregivers or partners/spouses in the PFAC and A‐LiNK to contribute to the resource. We sought out and received three writing contributions from caregivers who identified as fathers with the ICN and A‐LiNK networks, and these were added to the resource. Additionally, we reflected on the limited racial and ethnic diversity of our team and sought a contribution from a caregiver of color who contributed to the resource anonymously per the caregiver's request. We edited the formatting for each section to have a broad introduction, quotes from caregivers and psychosocial clinicians, and then action items (e.g., “What Can I Do Today”) with review of reputable, evidence‐based resources (e.g., video links to diaphragmatic breathing as a relaxation tool, links to the APA Division 54's Caregiver Wellbeing Special Interest Group's resources). Our QI team presented on the development of this caregiver coping‐focused resource and to solicit feedback before the resource was finalized at ICN's Fall 2024 Community Conference in Baltimore, MD in September 2024; our team received strong enthusiasm and excitement for the resource without additional constructive feedback.

Following the opportunity by all QI team members to review the caregiver coping resource draft, the text content was finalized in October 2024 and shared with the ICN Communications team to support formatting the resource. The caregiver coping resource was formally published on ICN's website in May 2025 with a creative commons license so this resource can be freely used and shared; the full resource can be found at https://www.improvecarenow.org/caregiver_coping_resource.

### 2.3 Ethical Considerations

QI team membership was relatively homogeneous, especially with respect to the roles of participating caregivers (i.e., all mothers). Given differences in coping between mothers and fathers of youth with IBD [10], we sought additional input from three caregivers who identify as fathers. Similarly, participant characteristics (i.e., all English‐speaking) and lack of funding precluded publication of the current resource in languages other than English. Further, to protect the confidentiality of any youth with IBD/AILD, caregivers who referenced information about their child refrained from including identifying information (e.g., name, age) and no photographs of any QI team members or caregivers' children were included, especially as youth and young adults of QI team members were not asked to provide documented assent or consent to include their protected health information. Per established IRB policies at the first author's institution when this work occurred (Nationwide Children's Hospital), this work was identified to be quality improvement and was exempt from IRB submission.

## 3 Results

### 3.1 Results

The caregiver coping resource QI project began through collaboration fostered by LHNs where caregiver and psychosocial stakeholders knew one another and this engendered the ability to share ideas about a caregiver‐focused resource. Caregivers and psychosocial clinicians used QI methodology to collaboratively work together nationally and via two LHNs, ICN and A‐LiNK; the QI team communicated electronically and worked asynchronously to complete the caregiver coping resource content. Nearly 1.5 years after beginning the caregiver coping resource QI project, the full text was finalized in October 2024 and was fully formatted and made freely available to the ICN and A‐LiNK communities in May 2025.

The caregiver coping resource begins with an introduction to the resource, including encouragement for caregivers to reach out to their child's healthcare team with any questions as they review the resource, followed by brief biographies of all caregiver and psychosocial contributors. The caregiver coping resource has the following sections: (1) Diagnosis, (2) Navigating the Joys and Challenges of Parenting a Child with IBD and/or AILD, (3) How Stress Impacts Our Overall Health, (4) Recognizing Emotions & Coping, (5) Fear & Anxiety, (6) Grief, (7) Guilt, (8) Medical Traumatic Stress, (9) Parental Coping and Self‐Care, (10) Taking Action, (11) The Male Caregiver Perspective, (12) Navigating Health Conditions & Siblings, (13) Being a Role Model For Your Child Navigating and Coping With Feelings, (14) Talking About Emotions & Coping with Your Child, (15) Seeking Peer Support for Your Child, (16) Checking in with Your Child—Knowing When & When Not to Push, (17) Supporting a Healthy Body Image and Food Relationship in the Context of Chronic Illness, (18) Advocating for your child in and out of the medical setting, (19) Teaching Developmentally Appropriate Self‐Advocacy Skills, (20) The Impact of a Chronic Illness on Siblings & the Family Unit, (21) Continuing to Implement Life/Home Expectations & Learning Balance, (22) Special Considerations in IBD (Very Early Onset Inflammatory Bowel Disease; Surgery for IBD; Special Consideration in AILD), (23) Co‐Morbid (Co‐Occurring) Diagnoses, (24) Health Equity, (25) Practical Tools & Suggestions Navigating Life and Pediatric Chronic Illness, (26) Chronic Illness Burnout and Treatment Plan Challenges, (27) How to talk about your child's IBD Diagnosis, (28) Handling work while caring for a sick child, (29) Insurance & Finances, (30) Travel, and (31) Key Takeaways.

Broadly, the caregiver coping resource is broken down into various topical areas including caregiver coping (e.g., stress, medical trauma, guilt), caregiver support of child coping (e.g., talking about emotions, peer support, advocacy, siblings), special considerations within IBD and AILD (e.g., surgery in IBD), and logistical topics (e.g., working with a sick child and navigating insurance coverage). Each section begins with an overview to introduce the topic, followed by caregiver and psychosocial clinician quotes, and lastly key takeaways including reputable resources; the resource pages include a QR code to a web‐paged collection of the links so that caregivers provided with hard copies of the resource can easily access the resources and caregivers provided with electronic copies can easily access these same resources through clicking hyperlinks. The completed, formatted resource totals 164 pages and is freely available on ICN's website as of May, 2025 and can be viewed by visiting https://www.improvecarenow.org/caregiver_coping_resource. In addition to the full 164‐page resource, a one‐page handout was developed with a QR code to the full resource to facilitate increased likelihood that clinicians will print the one‐page handout to ultimately increase accessibility of sharing with caregivers in clinical settings; the one‐page handout can also be found on the caregiver coping resource website (listed above). Announcements about the caregiver coping resource release were announced via official ICN listserv emails (which include clinicians, caregivers, and patients who have signed up for ICN emails) and ICN social media accounts in May, 2025. This process of announcing the resource and having the resource available on ICN's website follows the standard practice for other ICN community‐developed resources.

As an illustrative example, the Health Equity section begins with an introductory paragraph that reads, “While all children and families deserve equitable, compassionate, and timely care, we know from research and lived experience that experiences receiving and seeking care can feel very different depending on who your child is and where your child lives.” An anonymous caregiver contributor's quote then reads, “It is critical for healthcare professionals to have cultural sensitivity and humility training, and to never underestimate the power of really listening. There are unfortunately dangerous historical stereotypes that exist in healthcare, such as that Black/African American people and other people of color do not feel pain or can tolerate/endure more pain than others. My young child was subjected to horrendous treatment with adult‐sized needles, repeated blood draws, procedures/treatment that made my child very sick and engendered medical traumatic stress that was minimized, dismissed, reduced to my child being anxious when in truth, my child was in significant pain. We changed our child's IBD team three times, and ultimately found the right fit with a private practice GI and pursued patient advocacy before finally having our voices heard and our child's needs met! Patient support, resources, accessible patient advocates, culturally responsive medical staff and social workers need to be built in especially for patients of color and linguistically diverse families. Additionally, inpatient care should have inclusive resources for all patients, such as large‐toothed combs and/or brushes for curly hair and shampoo, conditioner, and moisturizer for curly hair (our hospital did not have shampoo for our child's hair as a young person of color), and nursing staff need to be skilled at how to sensitively use the products and affirm each child's beauty and uniqueness.” The reputable resources that follow include organizations like the Color of Gastrointestinal Illnesses, South Asian IBD Alliance, and recommendations of how to share health equity concerns with a healthcare team.

## 4 Discussion

### 4.1 Summary

Prior to this QI initiative, there were no publicly available caregiver‐focused resources for pediatric IBD and/or AILD to the knowledge of these authors. Consequently, this resource gap did not positively support caregiver coping in the setting of complex pediatric‐onset chronic illnesses and presented an urgent need to build such a resource. This QI work sought to address this significant clinical need through (a) co‐producing a resource by and for caregivers of children with IBD and AILD to have such a resource to share with caregivers in clinical care, and (b) sharing our story about how our team worked together and used QI methodology to create a resource in the hopes of creating a “blue print” for other passionate LHNs to consider doing the same for their communities. The creation of this caregiver coping resource is novel in that the QI project required significant and longstanding collaboration between caregivers and psychosocial clinicians over 1.5 years, all fostered by LHNs that created opportunities for such collaboration. This resource development follows the tradition of historical ICN QI initiatives that have led to freely accessible and reputable resources being infused back into the LHN [3, 21, 22]. The caregiver coping resource is freely and publicly available to the ICN and A‐LiNK communities (https://www.improvecarenow.org/caregiver_coping_resource); our QI team is hopeful that this resource will provide depth and breadth in supporting caregiver coping and further the mission of addressing the caregiver resource gap in IBD and AILD. While this is an important and exciting chapter in the story of how passionate stakeholders came together via LHNs to create a resource needed by our communities, the story is not over yet as the future chapters of studying the impact, acceptability, and effectiveness of this resource lay ahead.

### 4.2 Limitations

Despite multiple strengths of the project, we recognize inherent limitations. First, QI team members self‐selected to volunteer, resulting in selection effects that affected the generalizability of caregiver and psychosocial clinician perspectives. In particular, caregivers were not representative of all caregivers of youth with IBD/AILD based on racial, ethnic, spiritual, socioeconomic, gender identity, and/or sexual orientation identities. Similarly, caregivers who already engage in condition‐specific advocacy and support programs (e.g., ICN, A‐LiNK) may not reflect the backgrounds, experiences, and psychosocial needs of all caregivers of youth with IBD/AILD. Incorporating more diverse caregiver perspectives and expanding the resource to translations in languages other than English are areas of growth for future iterations of the resource. Additionally, the four psychosocial clinicians were 75% White and 25% Asian, and this composition does not present GI psychosocial clinicians and may impact generalizability. Seeking out diverse psychosocial clinician voices will also be a key consideration for future resource generation.

The caregiver coping resource is accessible via the freely available ICN link (https://www.improvecarenow.org/caregiver_coping_resource), downloading or viewing the PDF document, or printing hard copies of the resource. This introduces potential challenges for caregivers to access the resource; for example, caregivers may be unaware of the resource unless they seek it out online or receive a specific recommendation by their GI care team. This also requires reliable internet access, although most adults within the United States have consistent use of the internet, a smartphone, and/or high‐speed internet at home [30]. In the future, it would be helpful to create a fully web‐based version of the resource to improve the viewability on a smartphone. Our team worked to be as mindful and inclusive as possible with the constraint of not having funding for this QI project to support a fully web‐paged platform; as noted above, the section resource pages include a QR code that directs users to a web‐based list of links so that caregivers given hard copies of the caregiver coping resource can still easily access the links while caregivers using the PDF version can click on the hyperlinks. Additionally, our team created a one‐page handout containing a summary of the resource and a QR code to the full 164‐page resource to promote easier sharing of the resource by reducing printer burden; stated differently, we are hopeful that ICN and A‐LiNK clinicians will find printing one‐page handouts about the caregiver coping resource more feasible in clinical practice than printing copies of the 164‐page resource.

Our team does not yet have qualitative or quantitative outcomes data for caregivers of children with IBD and/or AILD who will use this resource in the future. Such outcomes data will be essential for identifying strengths and weaknesses of the existing resource and informing improvements to future iterations. Future work to assess the lived experiences of clinicians recommending the caregiver coping resource and caregivers using the resource and outcomes associated with use of this resource is needed and should integrate mixed‐methods and longitudinal methodology. Quantitative data should include tracking of click rate data to the caregiver coping resource landing page as well as the specific resource over at least 1 year to understand if use of the resource spikes in correlation to ICN communication efforts to announce the resource or if the resource use is stable over time. ICN and A‐LiNK site leads should be surveyed to understand how, if at all, this resource was integrated into clinical care (e.g., giving the one‐page handout in the infusion clinic) as well as understanding any barriers to sharing this resource across the ICN and A‐LiNK networks, which will also provide perspective into regional and site size (e.g., small or large practices) descriptives. Caregivers who have reviewed at least one section of the resource (given the length and detail, the full resource may not be relevant to all caregivers) should have semi‐structured interviews to understand the qualitative experience of using the resource as well as completing measures related to anxiety, mood, and adjustment to their child's disease to understand if the resource is associated with improved mental health and coping for caregivers. Taken together, these proposed future evaluation strategies will give more color to if, how (e.g., hard copy versus PDF copy), when (e.g., at diagnosis), and where (i.e., evenly used across all ICN and A‐LiNK centers) the caregiver coping resource is being used will be critical to understanding the impact, equity, and clinical utility of this resource. Data from future evaluation will help to determine barriers to equity and accessibility to decide appropriate next research and QI steps as well as to identify relevant measures to capture concepts of equity and accessibility in coordination with ICN's Health Equity committee's expertise and guidance.

## 5 Conclusions

The caregiver coping resource is the product of close collaboration between caregivers of children with IBD and AILD and psychosocial clinicians. Highlighting caregiver voices and lived experiences is essential for the validity, generalizability, and supportive tone of the resource content. Psychosocial clinicians added to the resource by incorporating evidence‐based knowledge and recommendations as well as information gleaned from many years of direct patient care with children with IBD and/or AILD and their caregivers. While this resource is an important addition to existing IBD‐specific resources [3, 21, 23], we are hopeful that this work inspires similar efforts in other pediatric populations.

Although the resource is not intended as a replacement for caregiver‐focused behavioral health treatment, it begins to address current gaps in available psychosocial resources for caregivers of youth with IBD/AILD. From a health equity perspective, we are hopeful that this resource may also improve access to evidence‐based information and strategies to support caregivers' psychosocial functioning, as caregiver‐focused behavioral health interventions may be limited to certain caregivers depending on geographic location, socioeconomic resources, and institution‐based services [6].

While we hope the caregiver coping resource will have direct benefits for caregivers of youth with IBD and AILD, it is essential to consider dissemination methods to maximize access. GI clinicians, social workers, and psychosocial clinicians must first be aware of the caregiver coping resource; to increase awareness, the resource release date was announced at the Live Online Community Conference in April 2025, and the resource release was announced via the official ICN email listserv as well as through official ICN social media accounts. As noted earlier in the manuscript, we also created a one‐page handout with a summary of the caregiver coping resource and a QR code to the full resource to increase clinical dissemination of the resource to encourage clinicians handing out one‐page handouts versus handing out 164‐page resources. Through ICN communications, we have encouraged GI clinics to have printed copies of the handout and/or full resource that can be provided to caregivers during hospitalizations, medical clinic visits, and infusion clinic visits. The availability of the resource by sharing the website (https://www.improvecarenow.org/caregiver_coping_resource) was designed to increase the likelihood of clinicians sharing the resource through patient portals with caregivers. Further, caregivers can also have a significant impact on the dissemination of the resource by providing the hyperlink in social media groups and posts that reach a larger audience of caregivers who are often seeking additional information and support related to IBD and AILD.

## Funding

The authors have nothing to report.

## Conflicts of Interest

The authors declare no conflicts of interest.

## Supporting information


**Data S1:** Supporting Information.

## Data Availability

The data that support the findings of this study are available on request from the corresponding author. The data are not publicly available due to privacy or ethical restrictions.
