# Supplementary material for: The Development and Co‐Production of a Caregiver Coping Resource for Pediatric Inflammatory Bowel Disease and Autoimmune Liver Disease
Source: Learn Health Syst. 2026 Feb 17;10(2):e70061. doi: 10.1002/lrh2.70061 (PMC12912881; doi:10.1002/lrh2.70061)
Supplement: Supplementary file 1 — Data S1: Supporting Information. [file LRH2-10-e70061-s001.docx]

**Title:**

The Development and Co-Production of a Caregiver Coping Resource for Pediatric Inflammatory Bowel Disease and Autoimmune Liver Disease

**Authors:**

- Jennie G. David-Rodgers, Ph.D., Pediatric Psychology and Neuropsychology, Nationwide Children’s Hospital, Columbus, OH
- Christina E. Holbein, Ph.D., Psychiatry and Behavioral Sciences, Children’s Hospital of Philadelphia, Philadelphia, PA
- Hannah McKillop, Ph.D., Pediatric Psychology and Neuropsychology, Nationwide Children’s Hospital, Columbus, OH
- Maria E. Lester, Nemours Children’s Health, Orlando, FL; ImproveCareNow Learning Health System; Autoimmune Liver Disease Network for Kids Learning Health System
- Victoria Levine, Children’s Hospital of Philadelphia, Philadelphia, PA; ImproveCareNow Learning Health System
- Ildiko Mehes, Children’s Hospital of Philadelphia, Philadelphia, PA; ImproveCareNow Learning Health System
- Heidi C. Riechel, B.A., Children’s Healthcare of Atlanta, GI Care for Kids, Atlanta, GA; ImproveCareNow Learning Health System
- Jane R. Weyer, Cincinnati Children’s Hospital Medical Center, Cincinnati, OH; ImproveCareNow Learning Health System; Autoimmune Liver Disease Network for Kids Learning Health System
- ImproveCareNow Learning Health System
- Autoimmune Liver Disease Network for Kids Learning Health System

**Funding information/financial disclosures:**

The authors have no funding information or financial disclosures to report. There are no conflicts of interests for the authors.

**Acknowledgments:**

The authors would like to warmly acknowledge and thank the following individuals who contributed to the caregiver coping resource: Brenda Barranco, Chris Browner, Noelle Moore, and Erin Spaulding. The authors would also like to thank Sarah Nocito for her formatting support, including the mock-ups presented in this manuscript.

**Data Availability Statement:**

No new data was created. The study reflects quality improvement work and as such does not have data.

**Word Count:**

4885

**Tables:**

1

**Corresponding Author Contact Information:**

Jennie David-Rodgers, Ph.D.

Pediatric Psychologist

Nationwide Children's Hospital, 700 Children's Drive, Columbus, Ohio 43205

[jenniedavidphd@gmail.com](mailto:jenniedavidphd@gmail.com)

(Phone) 614-722-2627

(Fax) 614-722-4718
